# National Surveillance for *Clostridioides difficile* Infection, Sweden, 2009–2016

**DOI:** 10.3201/eid2409.171658

**Published:** 2018-09

**Authors:** Kristina Rizzardi, Torbjörn Norén, Olov Aspevall, Barbro Mäkitalo, Michael Toepfer, Åsa Johansson, Thomas Åkerlund

**Affiliations:** Public Health Agency of Sweden, Solna, Sweden (K. Rizzardi, O. Aspevall, B. Mäkitalo, T. Åkerlund); Örebro University, Örebro, Sweden (T. Norén);; Unilabs Clinical Microbiology, Skövde, Sweden (M. Toepfer);; Växjö Hospital, Växjö, Sweden (Å. Johansson)

**Keywords:** Clostridioides difficile, Clostridium difficile, CDI, surveillance, typing, epidemiology, outbreaks, antibiotic resistance, antimicrobial resistance, bacteria, bacterial infection, Sweden

## Abstract

We report results from a national surveillance program for *Clostridioides difficile* infection (CDI) in Sweden, where CDI incidence decreased by 22% and the proportion of multidrug-resistant isolates decreased by 80% during 2012–2016. Variation in incidence between counties also diminished during this period, which might be attributable to implementation of nucleic acid amplification testing as the primary diagnostic tool for most laboratories. In contrast to other studies, our study did not indicate increased CDI incidence attributable the introduction of nucleic acid amplification testing. Our results also suggest that successful implementation of hygiene measures is the major cause of the observed incidence decrease. Despite substantial reductions in CDI incidence and prevalence of multidrug-resistant isolates, Sweden still has one of the highest CDI incidence levels in Europe. This finding is unexpected and warrants further investigation, given that Sweden has among the lowest levels of antimicrobial drug use.

## Abstract 2

Improved hygiene measures in healthcare settings likely caused the sustained nationwide decrease in infection rates.

In a 1995 study assessing incidence of *Clostridioides difficile* infection (CDI) in Sweden, 5,133 cases were reported, corresponding to an incidence of 58 cases/100,000 inhabitants (*1*). After the initial reports of outbreaks of CDI in Europe associated with PCR ribotype 027 (RT027) in 2005 (*2*), a second incidence study was conducted in 2007, which showed that CDI incidence had increased to 90 cases/100,000 inhabitants (8,276 new cases). Based on recommendations from the European Centre for Disease Prevention and Control, a national surveillance program for CDI was initiated in Sweden 2009, aiming to monitor the apparent nationwide increase in CDI cases, detect trends and outbreaks, and determine the baseline incidence of CDI in the catchment areas of local clinical laboratories (*3*). The program, conducted by the Public Health Agency of Sweden, includes voluntary laboratory reporting of all new and recurring CDI cases as well as epidemiologic typing and susceptibility testing of isolates from clinical laboratories.

Countries in Europe have large variations in CDI incidence rates and distribution of prevalent PCR ribotypes, and the highest incidence rates occur in the northern countries, even though these countries have a low prevalence of RT027 (*4*,*5*). In addition, across Europe, a weak negative correlation has been observed between CDI incidence rates and cephalosporin use (*5*). Here we summarize results of the national CDI surveillance program in Sweden during 2012–2016, including CDI incidence rates, distribution of *C. difficile* types, known CDI outbreaks, and the effect of changes in diagnostic methods on reported CDI incidence.

## Methods

### Voluntary Surveillance Program

We collected epidemiologic case data through a voluntary reporting system, in which local laboratories reported the total number of CDI cases each week, including information of patients who had prior episodes of CDI within the previous 8 weeks. A new CDI case was defined as a patient with CDI with no prior diagnosis of CDI within the previous 8-week period. The reporting, which started during week 43 in 2009, also included catchment area (i.e., county) and sex and age of the patient. Initially, 16 of 28 laboratories reported CDI data, and by the end of 2011, all laboratories had joined the surveillance program. We collected denominator data by using a separate questionnaire, distributed yearly, which included the total number of tests and the number of positive samples per laboratory.

We performed epidemiologic typing and antimicrobial susceptibility testing twice a year on isolates collected during weeks 11 and 39. We chose these weeks arbitrarily because there was no reason to assume seasonal variation of CDI. We asked the local clinical laboratories to culture samples from all suspected CDI case-patients and to test the fecal samples and bacterial cultures by using that laboratory’s standard diagnostic algorithm. To ensure that all isolates were identified during the study weeks, all culture-positive *C. difficile* isolates (including toxin-negative and toxin-positive according to the local laboratory’s standard test algorithm) were sent to the Public Health Agency of Sweden for PCR ribotyping and antimicrobial susceptibility testing. Approximately 4% of all yearly cases are analyzed by this program. The laboratories also sent information stating their current diagnostic method and the test results for each isolate sent for testing.

### PCR Ribotyping

From week 1 in 2009 through week 11 in 2012, PCR ribotyping was gel-based, as previously described by Stubbs et al. (*6*), with minor modifications (*7*). From week 39 in 2012, we performed PCR ribotyping with capillary gel electrophoresis and analyzed results with BioNumerics 7.5 (Applied Maths, Sint-Martens-Latem, Belgium) (*8*). We conducted identification on the basis of the Cardiff–European Centre for Disease Prevention and Control strain collection and other known types. We gave new types the prefix “x” followed by a chronological number until the strain was typed by the reference laboratory at Leiden University Medical Centre (Leiden, the Netherlands).

### Antimicrobial Susceptibility Testing

We performed susceptibility testing by using the antimicrobial drugs recommended for treatment (i.e., metronidazole and vancomycin) and common antimicrobial drugs known to increase risk for acquiring CDI (i.e., moxifloxacin, clindamycin, and erythromycin). We tested all isolates by using Etest on *Brucella* agar, as previously described (*9*). The breakpoints for resistant isolates were epidemiologic cutoff values according to the European Committee on Antimicrobial Susceptibility Testing (EUCAST): metronidazole, >2 mg/L; vancomycin, >2 mg/L; moxifloxacin, >4 mg/L; clindamycin, >16 mg/L; and erythromycin, >2 mg/L.

### Statistical Methods

We analyzed county-level PCR ribotype diversity by using the Simpson reciprocal index, 1/D. We analyzed how switching diagnostic method affected positivity rates by using the χ^2^ test. We compared ecologic MIC distributions of *C. difficile* isolates in Sweden to EUCAST distributions by using the Wilcoxon rank-sum test.

## Results and Discussion

### Incidence of CDI in Sweden during 2012–2016

**Figure 1:**
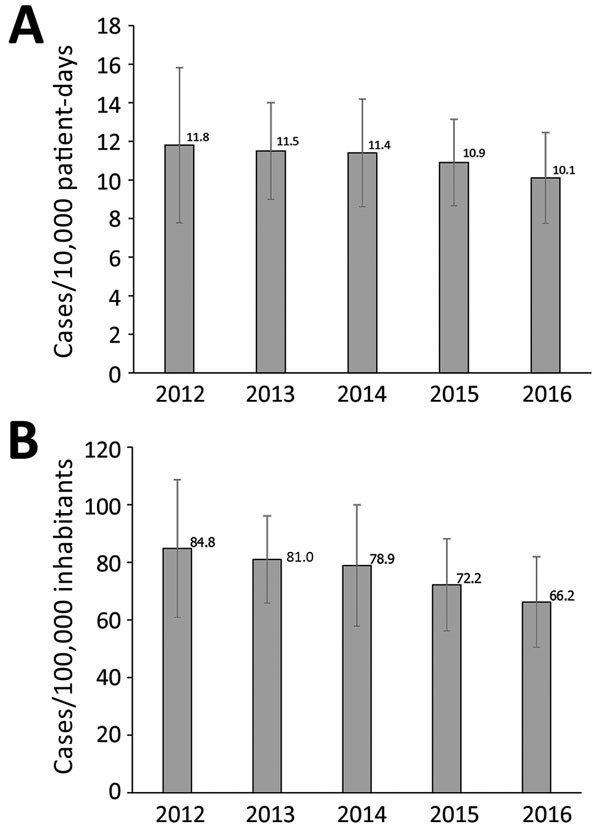
National incidence of *Clostridioides difficile* infection (CDI), Sweden, 2012–2016. A) CDI cases/10,000 patient-days. B) CDI cases/100,000 inhabitants. Error bars indicate SD of the mean county incidence for each year.

In 2012, a total of 10,820 cases of CDI were reported, of which 8,104 (75%) were new cases and 2,716 (25%) were recurrent cases. Recurrent cases represent a maximum estimate because the data included a few double samples from the same patient. The incidence of new cases was 11.8/10,000 patient-days and 85/100,000 inhabitants, results that were almost unchanged compared with those reported in 2007 (11.9/10,000 patient-days and 90/100,000 inhabitants). In 2016, the incidence of new cases had decreased to 10.1/10,000 patient-days (a 15% decrease) and 66/100,000 inhabitants (a 22% decrease) (Figure 1, panel A and B). The decrease occurred in most counties and resulted in less geographic variation in incidence (range 4.0–18.7/10,000 patient-days in 2012 compared with 7.1–15.5/10,000 patient-days in 2016 [Technical Appendix Figure]).

**Figure 2:**
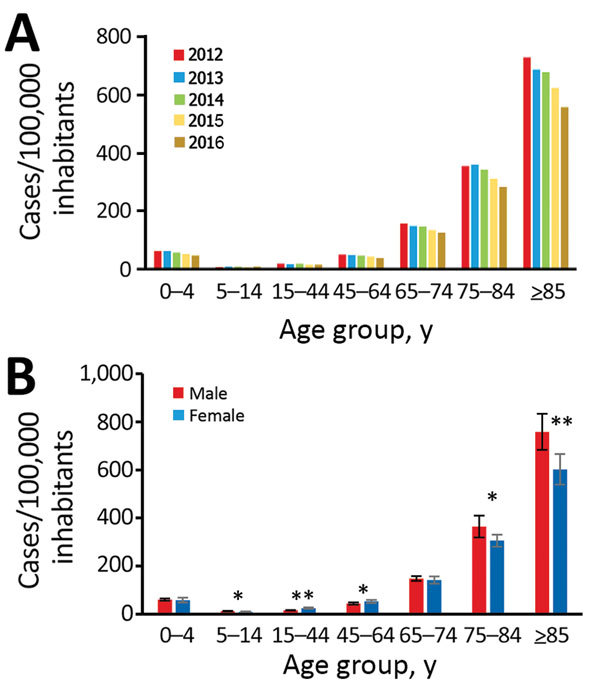
National incidence of new *Clostridioides difficile* infection (CDI) cases, Sweden, 2012–2016. A) Incidence by age group and year; B) incidence by age group and sex. Error bars indicate SD. *p<0.05; **p<0.01 (both by t-test).

CDI incidence decreased in all age groups except for 5–14 years; the largest reductions came in the age groups 0–4 years (25%), 45–64 years (23%), and >85 years (23%) (Figure 2, panel A). The incidence was reduced similarly in male and female inhabitants over time, although higher incidence occurred in male inhabitants 5–14 and >75 years of age during the entire period (Figure 2, panel B). For female inhabitants 15–64 years of age, higher incidence also occurred, 33% higher among the 15–44 years age group and 17% higher among the 45–64 years age group (Figure 2, panel B).

### PCR Ribotype Distribution

The distribution of the most common PCR ribotypes in Sweden has, with a few exceptions, been relatively constant since 2009 (Technical Appendix Table) and is comparable to that observed in other countries of Northern Europe (*4*). RT014 was the most common type throughout the whole period, except in 2011, when it was the second most common after RT020. Types frequently associated with multidrug resistance, such as RT012, RT078, RT046, and RT017, were among the 10 most common types in the first years of the surveillance program. In conjunction with the 7% reduction of incidence rate during 2014–2015, with the exception of RT078, all of the previously common multidrug-resistant (MDR) types were no longer among the 10 most common ribotypes. RT078 prevalence was ≈3% in Europe during 2012–2013; this ribotype is also common in pigs and calves (*4*,*10*). RT046 is predominant in scouring piglets in central parts of Sweden (*11*).

**Figure 3:**
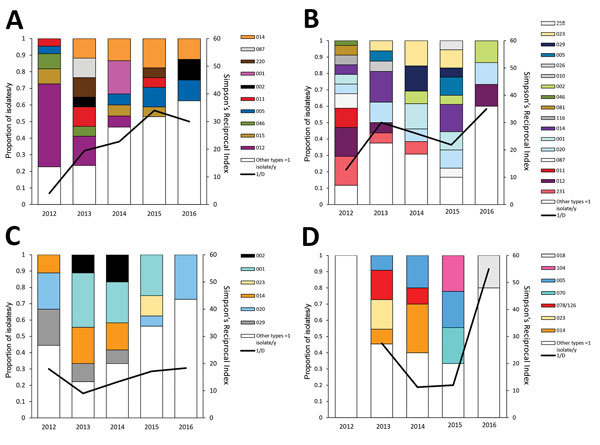
Variation in PCR ribotype distribution in 4 counties with large changes in *Clostridioides difficile* infection incidence rates, Sweden, 2012–2016. A) Östergötland; B) Uppsala; C) Västernorrland; D) Jämtland. 1/D, Simpson’s reciprocal index 1/D.

The distribution of PCR ribotypes was more variable between counties and over time. For example, Östergötland and Uppsala had relatively high levels of RT012 and RT231 in 2012 (Figure 3, panel A and B); these types are associated with outbreaks (*9*,*12*). By 2016, these types had diminished, and CDI incidence in these counties had decreased (to 35% in Östergötland and 52% in Uppsala) (Technical Appendix Figure). An increase occurred in PCR ribotype diversity over time (Figure 3, panel A and B). Similarly, a study in England indicated that ribotype diversity increased as outbreak-prone types decreased (*13*). These results (i.e., the disappearance of major types, increase in type diversity, and decrease in incidence) suggest that hospitals adopted improved infection control during the study period. In contrast, no change in ribotype diversity was observed in the county of Västernorrland despite an increased incidence during 2012–2015 (Figure 3, panel C). Jämtland was another county that showed strong incidence variation: an increase during 2012–2014 and then a decrease in 2016 (online Technical Appendix Figure 1). In Jämtland, we observed a change in ribotype diversity, from high diversity in 2012 to low diversity during 2014–2015, then back to high diversity in 2016 (Figure 3, panel D). However, no clustering of MDR PCR ribotypes was evident in Västernorrland or Jämtland. Possible explanations for the incidence levels might include polyclonal outbreaks, changes in diagnostics, and changes in sampling procedures. All 4 counties have changed diagnostic methods from enzyme immunoassay for toxin A and B (as standalone test) to generally more sensitive nucleic acid amplification tests (NAATs) (also as standalone test); Uppsala and Jämtland changed in 2012 and Östergötland and Västernorrland in 2015.

### Outbreaks of *C. difficile*

A geographic clustering of MDR isolates might be explained by a clonal outbreak that is not always apparent in clinical practice or in infrequent surveillance programs. One insidious outbreak caused by MDR RT046 was detected in 2011 in the Eksjö catchment area in Jönköping County (*14*), where an incidence of 22 CDI cases/10,000 patient-days surfaced in the national surveillance. The outbreak isolate was predominant in 46% of cases, and an excess virulence was observed by a 30-day mortality rate of 30% and a >40% recurrence rate compared with other ribotypes (T. Norén, unpub. data). Because the geographic clustering of RT046 in Jönköping was obvious already in 2008 (*3*), the impact of this outbreak cannot be fully understood. Antimicrobial drug stewardship and improved hygiene were implemented stepwise during the initial 10 months, but the outbreak was not controlled until chlorine disinfection was introduced after this period. In addition to RT046, significant (p<0.001) geographic clustering of RT231 was detected in 2008 in the counties of Stockholm and Uppsala (*3*), and this type spread between several hospitals during an extended period until it finally diminished (*8*).

Another outbreak, caused by toxin A–negative MDR RT017, was detected late in 2012 at Ystad Hospital in Skåne County. Considerable clinical impact occurred, similar to outbreaks with this type in other countries in Europe (*15*,*16*). During a 6-month period, 27 patients experienced severe CDI with this ribotype, and 10 died in spite of treatment. In November 2013, a hospital outbreak of RT027 CDI started at Växjö Hospital in Kronoberg County and was discovered when isolates from several fulminant cases were ribotyped. During August 2013–April 2016, a total of 41 patients had RT027 CDI diagnosed; 6 patients died, resulting in a 15% 30-day mortality rate for this strain. The strain was traced back to a patient that had been abroad, but whether his case was the actual index case was unclear. Because of the limited periods of strain collection in the national surveillance program, the smaller outbreaks of RT017 and RT027 CDI could only be traced retrospectively, and the outbreak alert in these cases was prompted by clinical awareness of clustering of severe cases.

Increased local incidence of CDI can be polyclonal, like in Jämtland County, where a sudden increase from ≈2–4 cases/week to 10–20 cases/week occurred during a few weeks of the 2013–14 winter season. Typing revealed 5–6 different susceptible PCR ribotypes, and although no clear evidence of transmission could be found, the sudden increase contributed to a substantially higher incidence in the county in 2013 and 2014 (Technical Appendix Figure 1). Increases of CDI in these scenarios might occur as a result of changes in diagnostic performance, in antimicrobial drug use, overcrowded wards, or a general decline in hygiene precautions, as opposed to introduction of a single virulent type.

### Sampling and Diagnostic Algorithms

**Figure 4:**
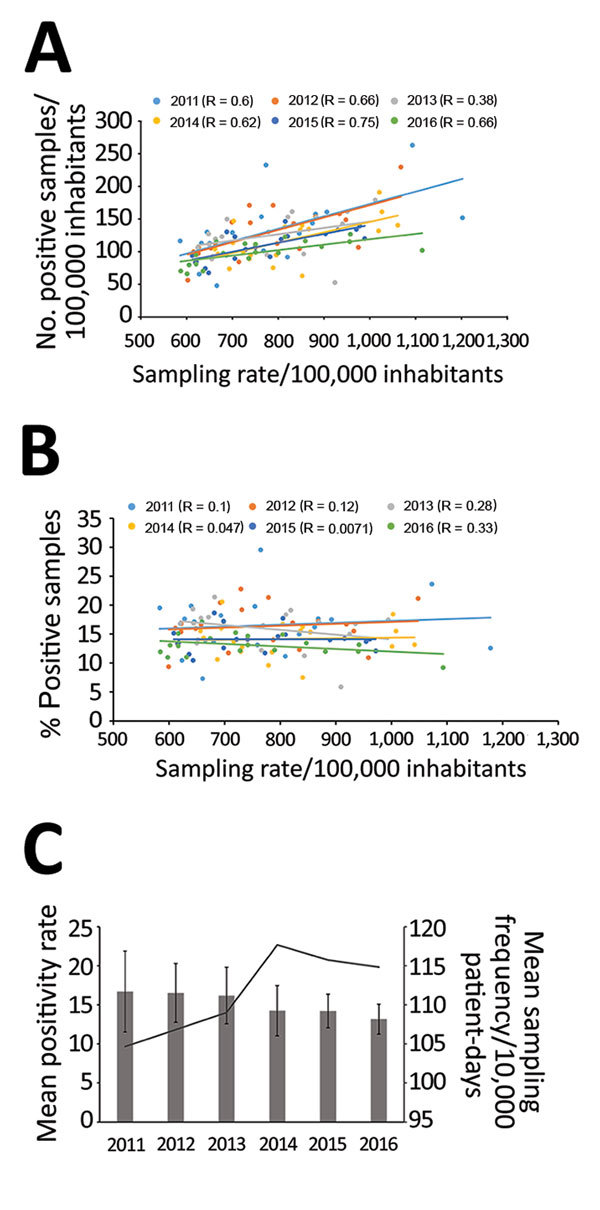
Correlation between *Clostridioides difficile* infection (CDI) cases and sampling rates, Sweden, 2009–2016. A) Correlation between number of positive CDI cases/100,000 inhabitants per county and sampling rates. B) Correlation between percentage of CDI cases and sampling rates. Dots indicate values per county; lines indicate regression analyses (R values as indicated). C) Mean positivity rate (bars) and mean sampling frequency (line), by year. Error bars show interlaboratory SD in positivity rates.

We found a positive correlation between sampling rate and CDI incidence per 100,000 inhabitants per county for all 6 years (Figure 4, panel A), consistent with findings in other studies (*17*,*18*). Indications for sampling and laboratory testing might differ between regions, and an extensive sampling might lead to lower positivity rates. However, sampling rates did not largely affect the diagnostic positivity rates, suggesting that the indications for sampling were similar among counties (Figure 4, panel B). The positivity rate and the intercounty variation in positivity rate decreased gradually during 2012–2016 (Figure 4, panel C), and, because many laboratories changed diagnostic methods during the period, the reduction in intercounty variation is most likely attributable to optimization of diagnostic algorithms and methods.

The number of laboratories using NAAT as a standalone method for CDI diagnostics increased from 6/28 in 2011 to 16/26 in 2016. Switching to NAATs as a stand-alone test has been associated with higher diagnostic sensitivity and up to a 67% increase in incidence (*19*–*21*), but we did not find any elevated positivity rate attributable to the increased use of NAAT. On the contrary, the average positivity rates 2 years after a method switch from enzyme immunoassay to NAAT decreased by 7% (Table). Moreover, we observed no consistent associations between method switch, positivity rate, and incidence. One explanation might be that most laboratories already had high sensitivity in the testing algorithms. Also, improved infection control and the elimination of certain PCR ribotypes that clustered in geographic areas during the study period most likely contributed to the lower positivity and incidence rates. Only 1 laboratory (laboratory 9, which serves Västerbotten County) showed a major increase in positivity and incidence rate after adopting NAAT after switching in 2013. The most likely explanation for this increase is that the laboratory used an unusual diagnostic method prior to the switch, including pretreatment of feces with alcohol followed by cell-cytotoxicity assay. A suboptimal diagnostic method might explain why this county had the lowest incidence rates in Sweden 2012 (Technical Appendix Figure 1).

**Table:** Comparison of CDI positivity rates for clinical laboratories that switched methods during the 2009–2016 study period, Sweden*

Change in testing algorithm	Year algorithm was switched	Mean no. positive samples (mean positivity, %)		p value†	Change in positivity after switch, %	Local CDI‡ incidence change 2012–2016, %
		2 y before switch	2 y after switch			
EIA to NAAT						
Laboratory 1	2011	698 (21)	672 (19)	0.004	−9	−52
Laboratory 2	2011	176 (13)	355 (16)	<0.001	+24	−31
Laboratory 3	2011	101 (12)	168 (16)	<0.001	+36	−9
Laboratory 4	2011	635 (22)	506 (19)	<0.001	−13	+6
Laboratory 5	2012	322 (15)	312 (17)	0.013	+14	−27
Laboratory 6	2013	416 (22)	377 (18)	<0.001	−18	−34
Laboratory 7	2013	346 (16)	300 (12)	<0.001	−27	−28§
Laboratory 8	2013	176 (11)	272 (14)	<0.001	+25	−28§
Total		359 (18)	370 (17)	<0.001	−7	
Cytotox to NAAT						
Laboratory 9¶	2012	124 (7)	353 (20)	<0.001	+68	+67
Laboratory 10	2013	1,398 (13)	1233(11)	<0.001	−18	−9§
Total		761 (12)	793 (12)	0.358	−2	
Cytotox to EIA + GDH						
Laboratory 11	2014	294 (17)	273 (13)	<0.001	−22	−35
NAAT to EIA + NAAT						
Laboratory 12	2014	388 (19)	290 (14)	<0.001	−27	−44
EIA to EIA + NAAT						
Laboratory 13	2014	677 (17)	420 (13)	<0.001	−21	−35

*CDI, *Clostridioides difficile* infection; EIA, enzyme immunoassay; GDH, glutamate dehydrogenase assay; NAAT, nucleic acid amplification testing.  †By χ^2^ test. ‡CDI incidence/100,000 population. Local incidence data from before 2012 not available. §Data from >1 laboratory is included in local CDI incidence rates. ¶This laboratory used a nonstandardized cell cytotoxicity assay.

### Antimicrobial Resistance

**Figure 5:**
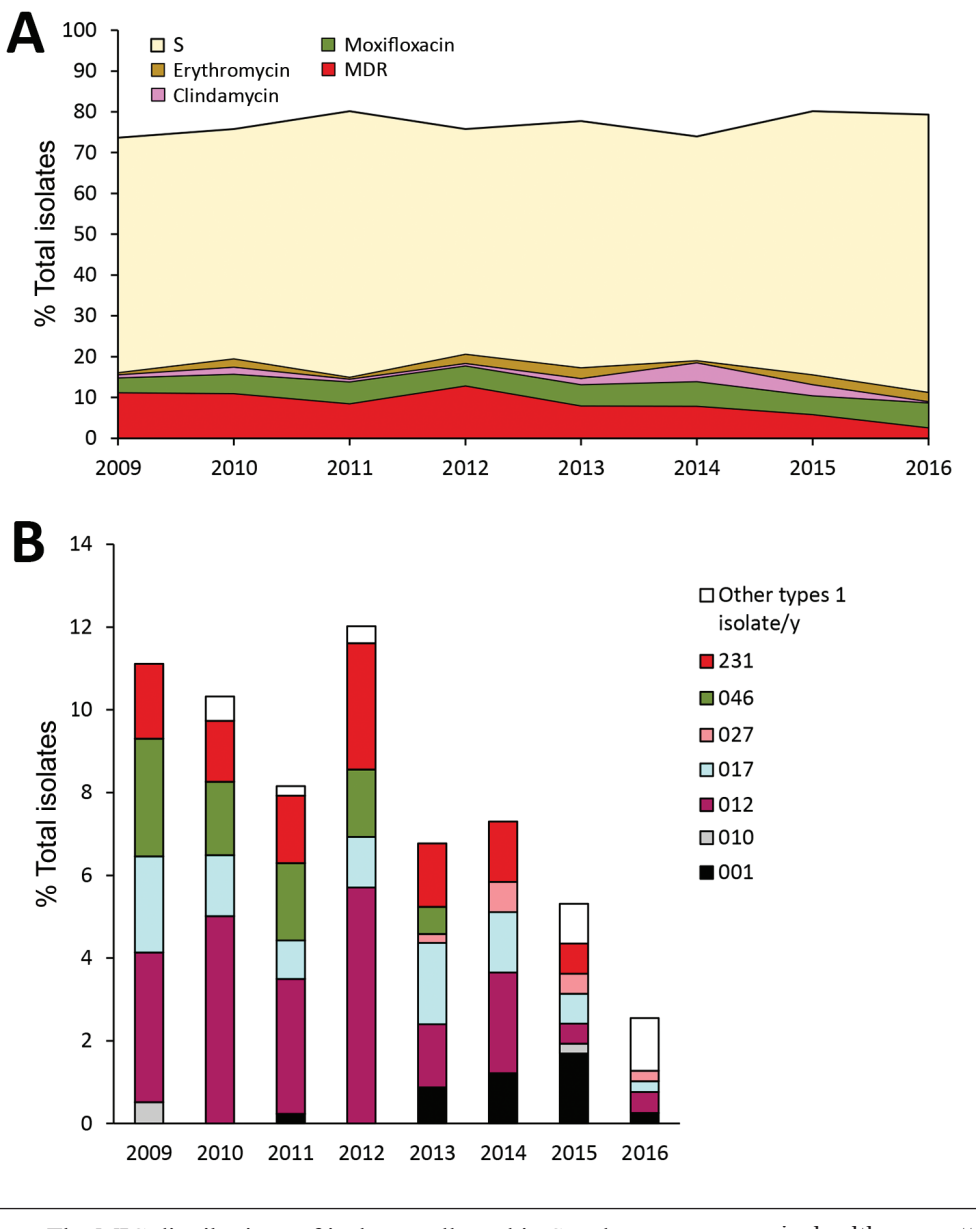
Resistance of *Clostridioides difficile* to indicator antimicrobial drugs, Sweden, 2009–2016. A) Percentage of isolates resistant and sensitive to indicator antimicrobial drugs erythromycin, clindamycin, and moxifloxacin. B) PCR ribotype distribution of MDR isolates. MDR, multidrug-resistant (i.e., resistant to erythromycin, clindamycin, and moxifloxacin); S, sensitive to erythromycin, clindamycin, and moxifloxacin.

All 3,321 isolates collected during 2009–2016 within the national surveillance program have been tested for antimicrobial susceptibility to vancomycin, metronidazole, erythromycin, clindamycin, and moxifloxacin. Only 1 isolate (RT027) was resistant to metronidazole (MIC 4 mg/L), and no isolate was resistant to vancomycin. The proportion of isolates resistant to erythromycin, clindamycin, and moxifloxacin was reduced during 2009–2016 (Figure 5, panel A). The highest proportion of resistant isolates was observed in 2012, when 13% of isolates were MDR (resistant to moxifloxacin, clindamycin, and erythromycin). During 2012–2016, the proportion of MDR isolates was reduced by 80%; moxifloxacin-resistant isolates were reduced by 26%, clindamycin-resistant isolates by 51%, and erythromycin-resistant isolates by 46%. During 2009–2012, from 94% to 97% of the MDR isolates belonged to 4 PCR ribotypes (RT012, RT017, RT046, and RT231). In 2016, the same ribotypes accounted for only 30% of all MDR isolates (Figure 5, panel B).

The MIC distributions of isolates collected in Sweden during 2009–2016 showed significant differences to those from EUCAST for all antimicrobial drugs tested except for clindamycin. The collection from Sweden had a greater proportion of isolates with lower MICs than did the EUCAST collection (p = <0.001 by Wilcoxon rank-sum test) (Technical Appendix Figure 2).

## Conclusions

During 2012–2016, a sustained decrease in incidence rates of CDI has occurred in Sweden, as well as a dramatic decrease in the proportion of MDR *C. difficile* isolates. Although decreased antimicrobial drug consumption or prudent use might be part of the explanation, we suggest that the major impact is attributable to improved hygiene measures in healthcare settings. This hypothesis is supported by 1) the fact that the volume of antimicrobial drugs typically associated with increased risk for acquiring CDI sold to hospitals, where CDI is predominant (*4*), was virtually unchanged during the study period (*22*); 2) a substantial reduction in CDI cases that occurred among elderly patients, who are known to be hospitalized to a greater extent; and 3) the apparent disappearance of geographic clusters of specific *C. difficile* PCR ribotypes, indicative of reduced nosocomial spread. However, because CDI cases are not classified into community- and healthcare-associated CDI, we cannot entirely rule out the possibility that the observed incidence reduction occurred mainly in the community, where antimicrobial drug sales have decreased more compared with sales to inpatient facilities (*22*). 

The surveillance system in Sweden has several limitations (e.g., the reporting is not mandatory, diagnostic methods vary across the country and over time, and isolates are collected only twice per year). However, the compliance of reporting has been rather high, probably because of the open reporting of information on geographic differences in incidence and clusters of *C. difficile* types. This reporting has in turn led to increased awareness of local epidemiology that is useful for tailoring hospital hygiene measures and antimicrobial stewardship policies. 

Despite the 22% decrease in CDI incidence during 2012–2016, Sweden still has a comparatively high CDI incidence compared with other countries in Europe. Because only a few outbreaks have been reported and diversity of types is high in northern Europe, including Sweden (*4*), the high incidence is probably not explained by nationwide outbreaks but is more likely attributable to increased clinical awareness, contributing to correct diagnoses and treatment. Sampling rates for CDI in Sweden are also high (*17*), an average of 116 samples/10,000 patient-days in 2016, a factor that is correlated with higher incidence (*19*–*21*). Introduction of highly sensitive methods such as standalone NAAT has been correlated with higher CDI incidence (*17*), but the small effect on positivity rates after adopting NAAT that we report suggests that previous diagnostic algorithms were on par with NAAT methods. A high incidence might also be related to more subtle differences in the population, such as immunity or susceptibility to CDI, a possibility that warrants further research.

Technical Appendix Ten most common PCR ribotypes detected, by year, county-specific incidence of *Clostridioides difficile* infection, and MIC distribution of collected isolates, Sweden, 2009–2016.
