# Supplementary material for: National Surveillance for Clostridioides difficile Infection, Sweden, 2009–2016
Source: Emerg Infect Dis. 2018 Sep;24(9):1617–25. doi: 10.3201/eid2409.171658 (PMC6106436; doi:10.3201/eid2409.171658)
Supplement: Technical Appendix — Ten most common PCR ribotypes detected, by year, county-specific incidence of Clostridioides difficile infection, and MIC distribution of collected isolates, Sweden, 2009–2016. [file 17-1658-Techapp-s1.pdf]

Article DOI: https://doi.org/10.3201/eid2409.171658

National Surveillance for *Clostridioides difficile* Infection, Sweden, 2009–2016

Technical Appendix

Technical Appendix Table. The 10 most common PCR ribotypes detected annually in Sweden, 2009–2016*

2009	2010	2011	2012	2013	2014	2015	2016
014 (12.9)	014 (12.4)	020 (10.5)	014 (14)	014 (9.4)	014 (10.7)	014 (11.1)	014 (10.5)
001 (7.2)	020 (10)	014 (8.2)	020 (9)	023 (7)	001 (8.3)	023 (8.2)	002 (10.5)
020 (7.2)	012 (7.7)	001 (5.6)	012 (6.9)	001 (5.9)	002 (6.6)	002 (7.5)	023 (6.4)
023 (5.5)	002 (7)	005 (5.4)	001 (6.1)	002 (5.2)	078 (5.6)	001 (7.3)	020 (6.2)
078 (5.2)	046 (5.6)	012 (5.4)	002 (5.3)	078 (4.6)	020 (5.4)	020 (6.3)	001 (4.9)
012 (5.2)	001 (5.3)	002 (5.2)	046 (4.3)	220 (4.4)	012 (4.4)	078 (5.3)	005 (4.6)
005 (4.4)	078 (5)	023 (4.9)	005 (4.1)	011 (4.2)	029 (3.7)	220 (4.8)	081 (3.8)
046 (3.9)	005 (4.1)	046 (3.7)	231 (3.7)	020 (3.7)	023 (3.7)	005 (4.4)	070 (3.6)
002 (3.6)	070 (3.2)	078 (3.5)	011 (2.7)	012 (3.5)	005 (3.4)	010 (3.6)	045 (3.3)
017 (3.4)	003 (2.7)	081 (3)	081 (2.4)	046 (3.3)	045 (3.2)	070 (2.7)	029 (3.3)

*The percentage for each type/year is shown in parenthesis.

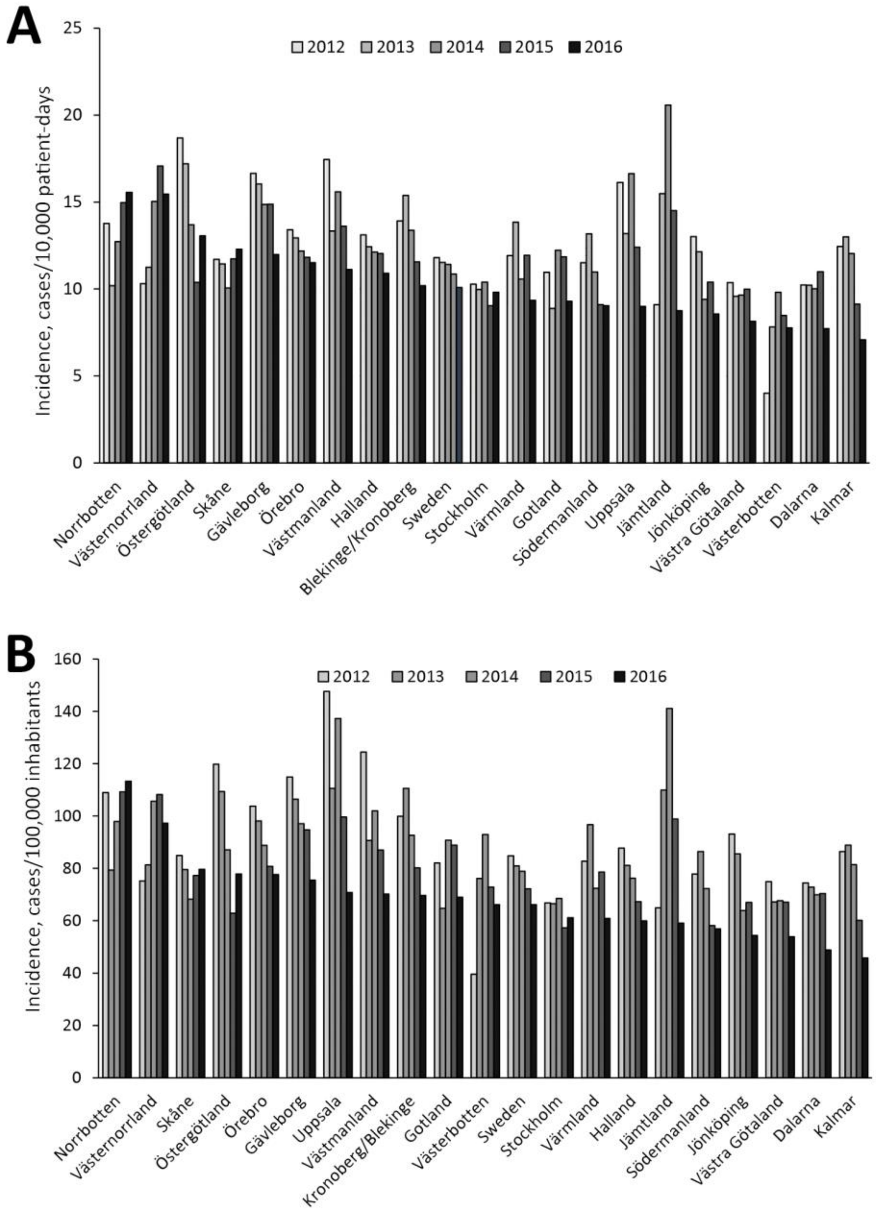

Technical Appendix Figure 1. County specific incidence of *Clostridioides difficile* infection (CDI). A) Incidence of CDI per 10,000 patient-days from 2012 to 2016. B) Incidence of CDI/100,000 inhabitants from 2012 to 2016.

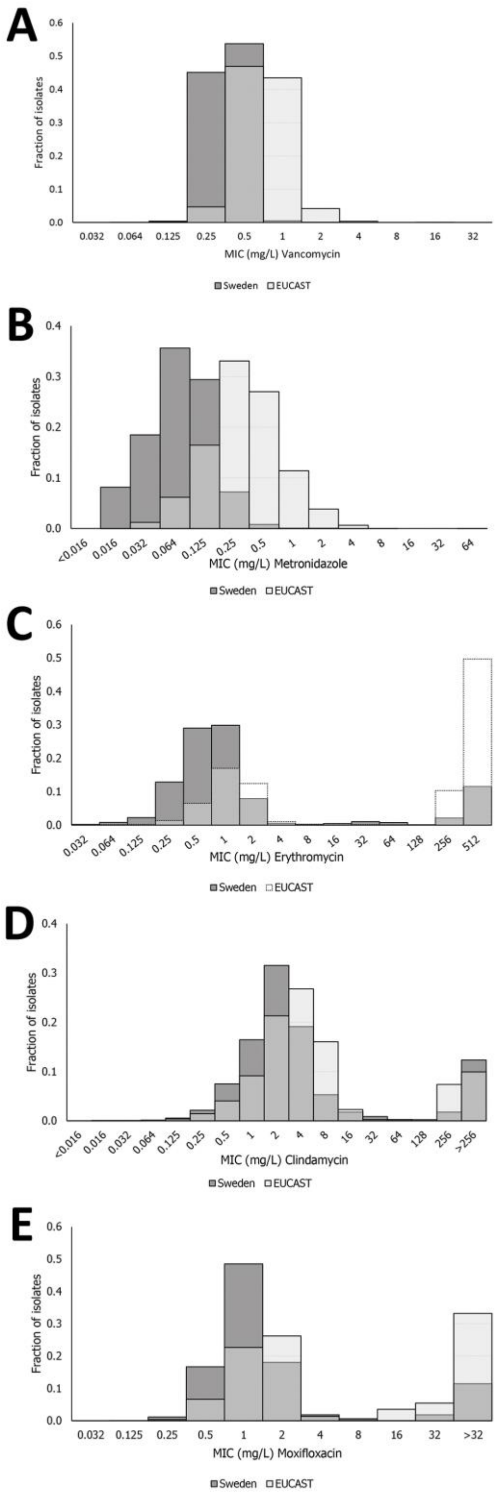

Technical Appendix Figure 2. MIC distribution of the Swedish isolates collected between 2009 and 2016 compared to the EUCAST collection. A) Vancomycin. B) Metronidazol. C) Erythromycin. D) Clindamycin. E) Moxifloxacin.
